# A Case of End-Stage Renal Disease With Hemifacial Swelling

**DOI:** 10.7759/cureus.42331

**Published:** 2023-07-23

**Authors:** Shubham Dubey, Pranjal Kashiv, Kapil Sejpal, Apoorvi Shah, Manish Balwani

**Affiliations:** 1 Department of Nephrology, Jawaharlal Nehru Medical College, Datta Meghe Institute of Higher Education and Research, Wardha, IND; 2 Department of Interventional Radiology, Jawaharlal Nehru Medical College, Datta Meghe Institute of Higher Education and Research, Wardha, IND

**Keywords:** end-stage renal disease (esrd), chronic kidney disease (ckd), balloon angioplasty, ct venogram, arteriovenous (av) fistula, access recirculation, central vein stenosis

## Abstract

A 64-year-old male, with end-stage renal disease on maintenance hemodialysis twice a week for the last two years, presented with swelling over the left half of his face, left side of the neck, and left upper limb for two months. The vascular access for hemodialysis was the left brachiocephalic arteriovenous (AV) fistula. There was no history of insertion of a dialysis catheter on the left side of the neck. Physical examination showed dilated and tortuous veins over the left side of his chest and left arm with normal-functioning AV fistula. Computed tomography (CT) venogram revealed narrowing in the left brachiocephalic vein and cephalic vein with multiple collaterals in the left upper limb and shoulder region. Ballon angioplasty was done across the stenotic segments, and a good flow was achieved with no remnant stenosis. This is a rare presentation as there was no history of cannulation of left-sided central vessels.

## Introduction

Patients on maintenance hemodialysis need a long-term vascular access with the least complications. The complications that can occur with vascular access are stenosis, thrombosis, digital ischemia, infection, heart failure, aneurysm, and pseudoaneurysm. In patients on maintenance hemodialysis, central vein stenosis (CVS) is primarily attributed to an ipsilateral central venous catheter placement, and its occurrence on the opposite side is seldom reported in the literature. Hence, we report this rare case of left CVS despite no previous cannulation on the left side.

Cannulation of the central veins lead to intimal injury, which is associated with increased smooth muscle cells and focal endothelial denudation [1]. CVS can lead to dreaded complications, such as access recirculation leading to inadequate dialysis, increased blood loss post dialysis, and vascular thrombosis ultimately leading to fistula failure [2].

## Case presentation

We present a case of 64-year-old male with end-stage renal disease and was on maintenance hemodialysis twice weekly for the last two years. The patient’s vascular access was the left brachiocephalic arteriovenous (AV) fistula, with no history of insertion of a left internal jugular dialysis catheter. He presented with swelling over the left half of his face, left side of the neck, and left upper limb for two months. The patient was initiated on hemodialysis two years back through a right uncuffed internal jugular dialysis catheter. Meanwhile, he underwent creation of a left radiocephalic AV fistula, which was a primary failure. Subsequently, the right uncuffed internal jugular dialysis catheter was removed, and a right uncuffed femoral dialysis catheter was placed. Henceforth, he underwent left brachiocephalic AV fistula creation, through which he is undergoing dialysis currently. There was no history of any intervention of AV fistula prior to this presentation.

**Figure 1:**
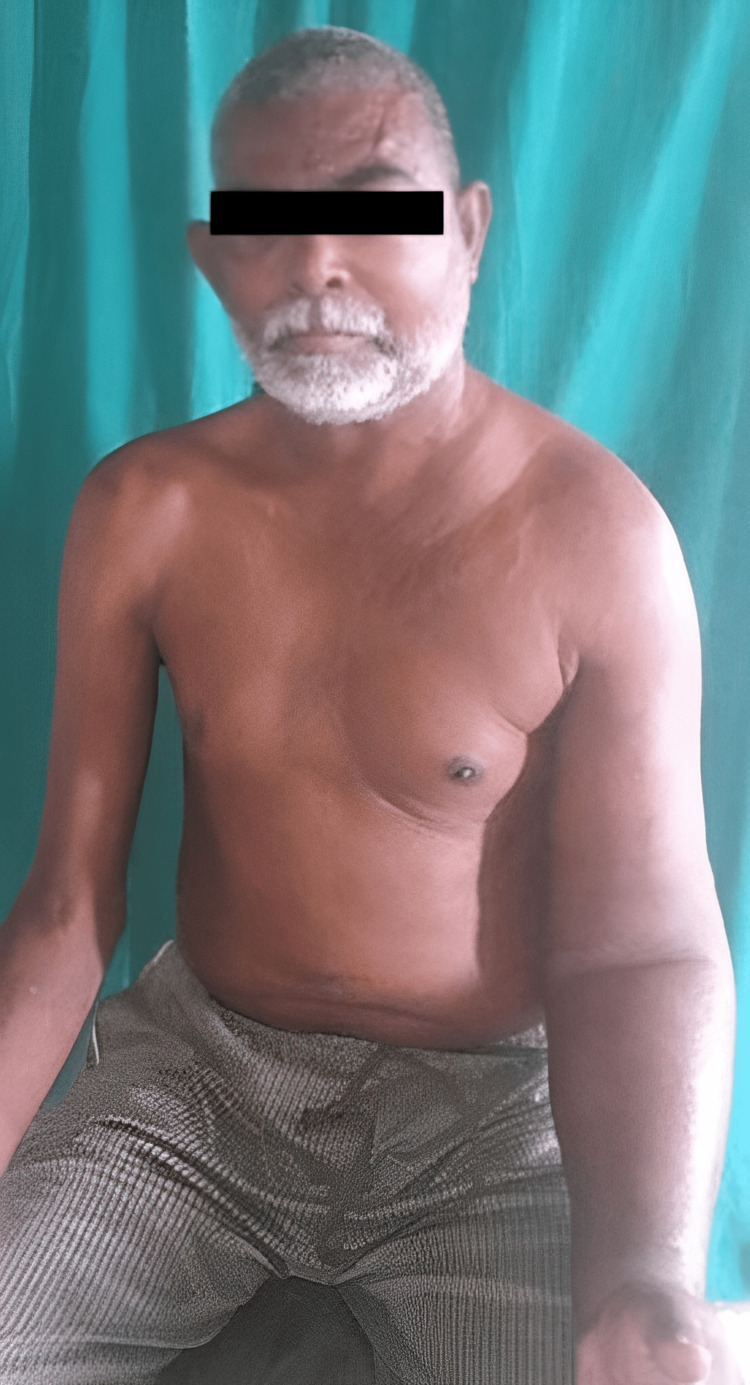
Clinical picture of the patient before balloon angioplasty

On examination, there was non-pitting edema over the left half of his face, neck, and left upper limb, with dilated veins over the left half of the chest and left shoulder region (Figure 1).

**Figure 2:**
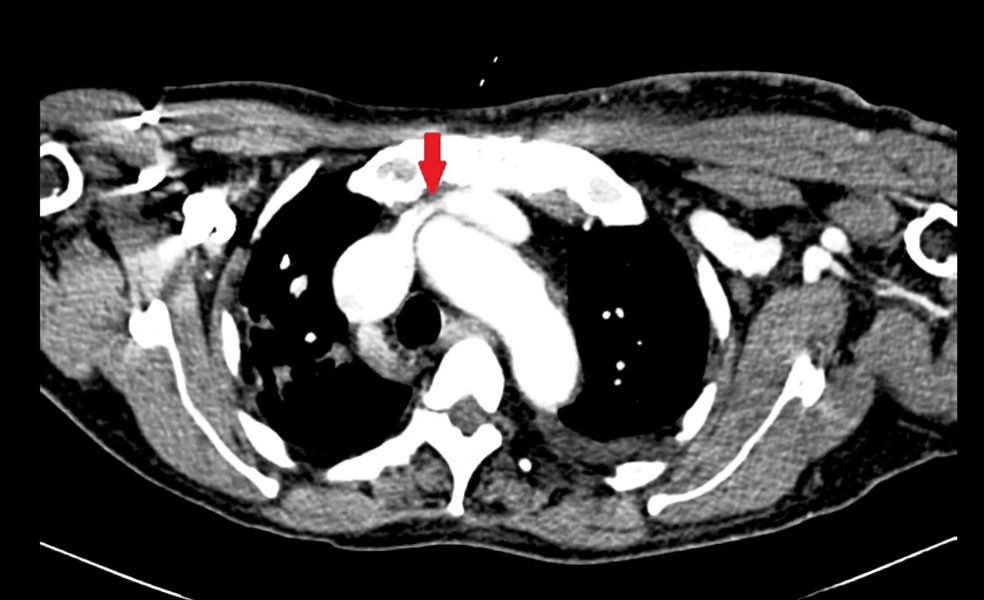
CT venogram showing narrowing of the left brachiocephalic vein (red arrow)

Color Doppler of the left upper limb was suggestive of brachial and cephalic vein dilatation without any stenosis or stricture. Computed tomography (CT) venogram revealed narrowing in the left brachiocephalic vein with multiple collaterals in the left upper limb and shoulder region (Figure 2).

**Figure 3:**
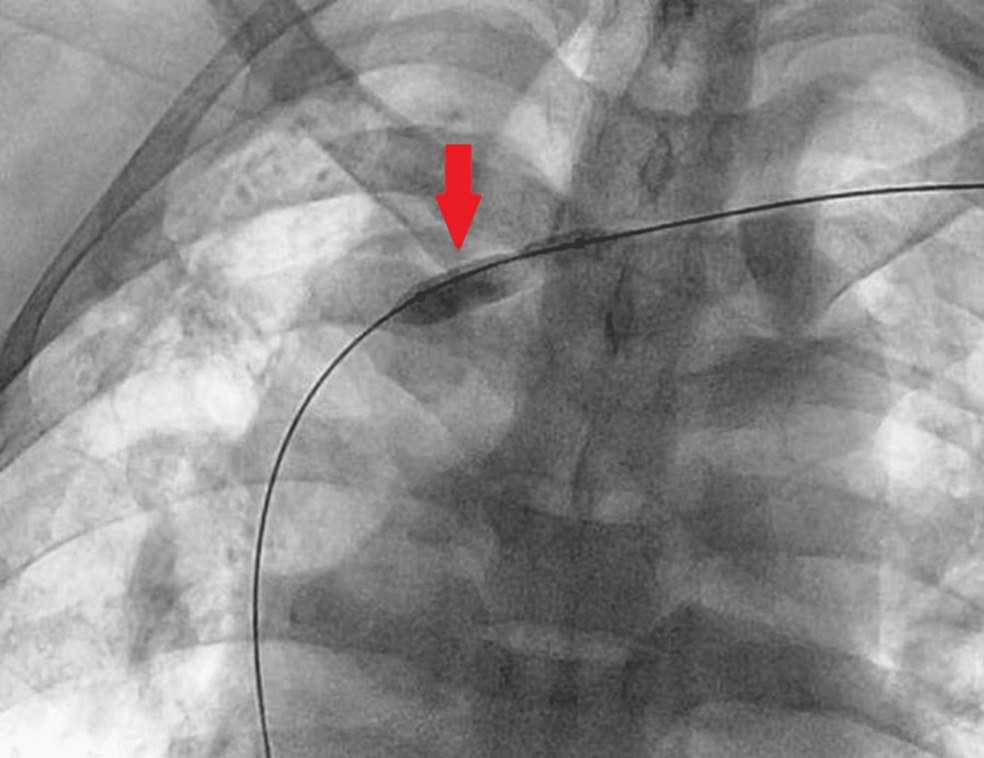
Balloon angioplasty of the left brachiocephalic vein (red arrow)

**Figure 4:**
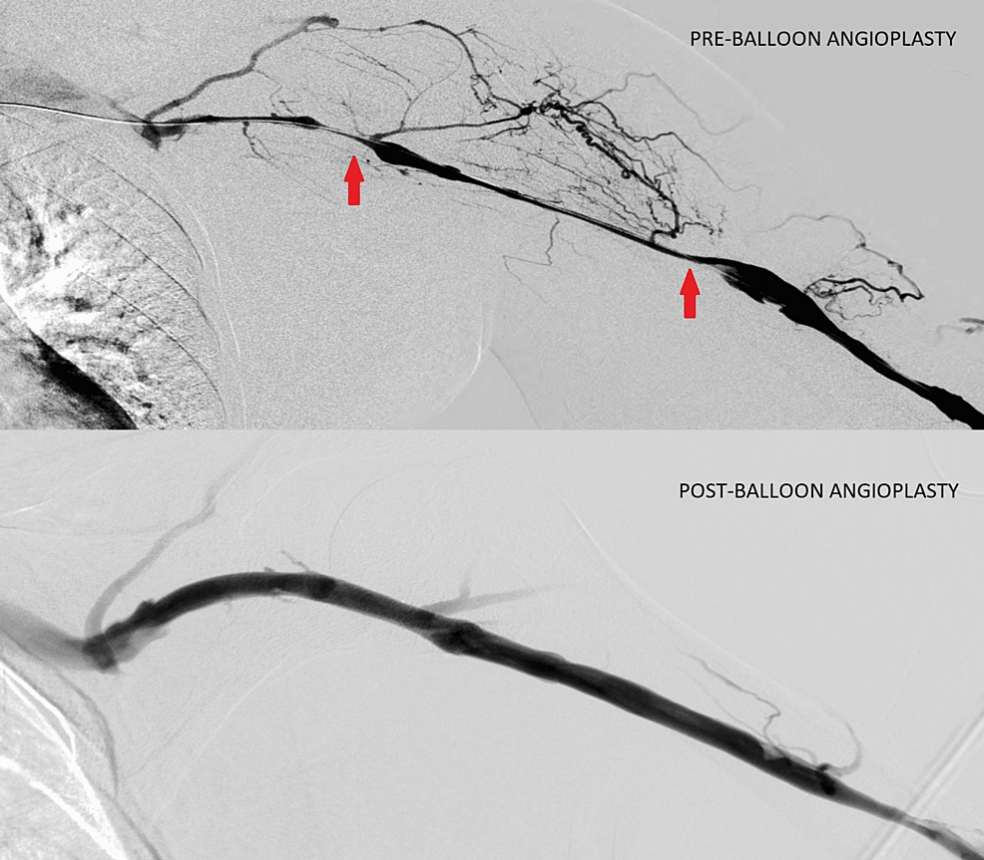
Pre- and post-balloon angioplasty of the left cephalic vein (red arrows showing stenotic segments of the left cephalic vein)

Balloon angioplasty was done across the stenotic segment of the left brachiocephalic vein (Figure 3) and left cephalic vein (Figure 4) using a 14x40 mm balloon and 8x60 mm balloon, respectively.

**Figure 5:**
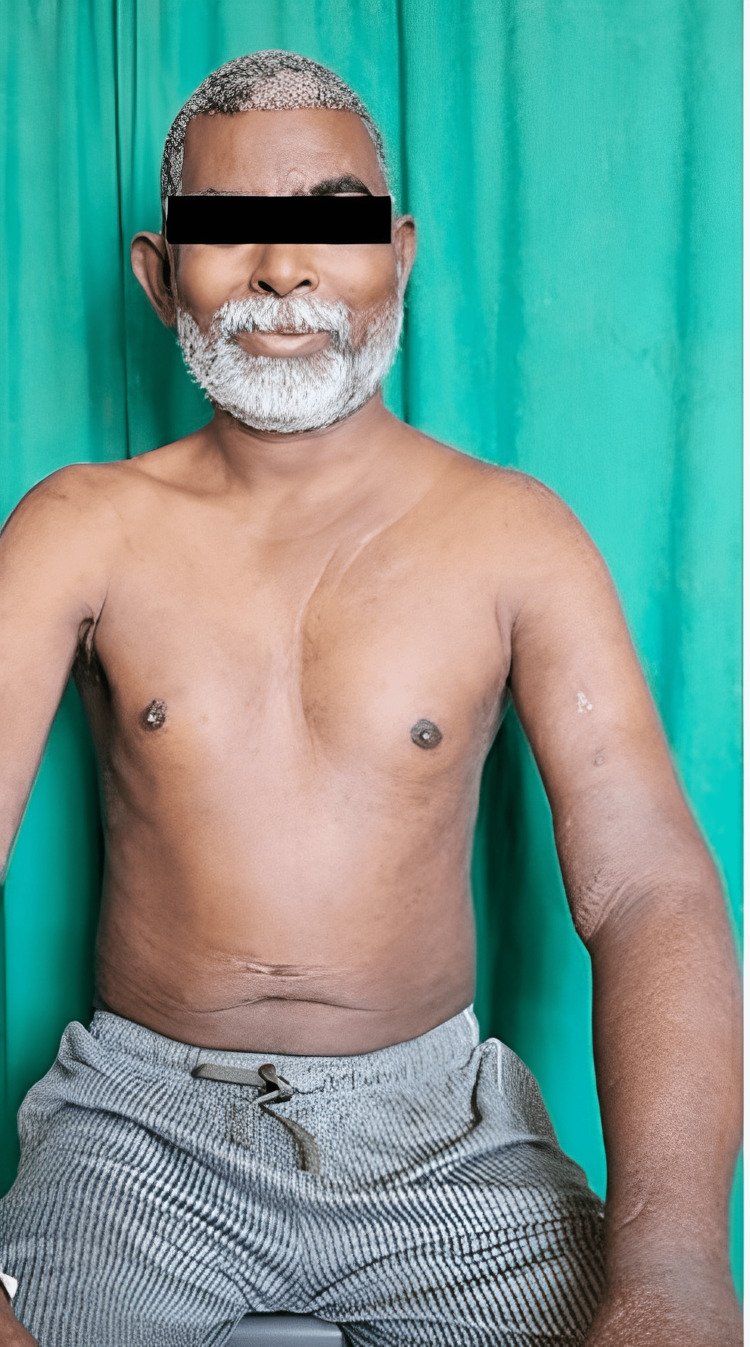
Post balloon angioplasty day two

Post balloon angioplasty venogram showed a good flow across the brachiocephalic and cephalic veins and no remnant stenosis at the origin. The swelling eventually subsided post procedure in a few days (Figure 5).

## Discussion

A central vein is a major vein that is close to the center of the blood circulation. Central veins include the superior vena cava, inferior vena cava, brachiocephalic veins, subclavian veins, common iliac veins, and external iliac veins [3]. Incidence of CVS ranges from 5% to 50% [4]. Pathogenesis of CVS involves endothelial injury due to central vein cannulation followed by inflammation and fibrosis. The risk differs on the basis of the vein cannulated. It is maximum with subclavian vein cannulation [5]. Patients with CVS usually present with swelling of the ipsilateral arm and face and may encounter issues, such as inadequate dialysis due to an increase in access recirculation. Moreover, stasis because of the stenosis can lead to thrombosis of the AV fistula due to the sluggish blood flow, causing AV fistula failure. In cases of prolonged CVS, patients may also complain of increased frequency of venous alarms from the dialysis machine or increase in blood loss following the removal of venous needle at the end of dialysis.

Treatments of CVS include percutaneous balloon angioplasty, angioplasty with stent placement, and newer techniques, such as hemodialysis reliable outflow (HeRO) graft [6,7]. Percutaneous transluminal balloon angioplasty (PTA) is the first-line treatment for central venous stenosis (CVS). The National Kidney Foundation Kidney Disease Outcomes Quality Initiative (NKF KDOQI) recommends percutaneous transluminal angioplasty (PTA), with or without stent placement; PTA is also considered the preferred approach to symptomatic CVS [7]. In cases of symptomatic CVS, endovascular treatments lead to initial success rates of 90%, but the long-term patency rates at six and 12 months are around 50% and 25%, respectively [5,8]. In view of the poor long-term patency with endovascular treatments, there was a need of alternate treatment options for CVS [8]. The HeRO Graft is a fully subcutaneous AV access option used for maintaining long-term access for hemodialysis patients having CVS. It is a graft, but it is different from a conventional AV graft in terms of having no venous anastomosis. It has shown to confer 87% cumulative patency at two years and 69% reduced infection rate compared with catheters. It has two primary components made of expanded polytetrafluoroethylene (ePTFE): an expanded arterial graft component and a venous outflow component.

## Conclusions

CVS is a dreaded complication of arteriovenous vascular access, which may lead to inadequate dialysis or graft loss. Avoiding cannulation of the central veins and creating an AV fistula well in advance before initiation of hemodialysis can prevent this complication. Treatments mainly include balloon angioplasty, but it has a very high risk of recurrence. Other newer techniques, such as the HeRO Graft, may be considered, but its cost and availability could be a challenge in developing countries, such as India.
